# Modulation of Multidrug Resistance Transporters by Food Components and Dietary Supplements: Implications for Cancer Therapy Efficacy and Safety

**DOI:** 10.3390/cimb46090576

**Published:** 2024-09-02

**Authors:** Agnieszka Brodzicka, Agnieszka Galanty, Paweł Paśko

**Affiliations:** 1 Department of Food Chemistry and Nutrition, Faculty of Pharmacy, Jagiellonian University Medical College, Medyczna 9, 30-688 Cracow, Poland; 2 Department of Pharmacognosy, Faculty of Pharmacy, Jagiellonian University Medical College, Medyczna 9, 30-688 Cracow, Poland; agnieszka.galanty@uj.edu.pl

**Keywords:** MRP2, BCRP, P-gp, natural products, dietary supplements, anticancer drugs, drugs carriers

## Abstract

The aim of this review is to explore how diet and dietary supplements influence the activity of key multidrug resistance (MDR) transporters—MRP2, BCRP, and P-gp. These transporters play a crucial role in drug efflux from cancer cells and significantly affect chemotherapy outcomes. This review focuses on how dietary phytochemicals, such as catechins and quercetin, impact the expression and function of these transporters. Both in vitro and in vivo experiments were examined to assess changes in drug bioavailability and intracellular drug accumulation. The findings show that certain dietary components—such as catechins, flavonoids, resveratrol, curcumin, terpenoids, sterols, and alkaloids—can either inhibit or induce MDR transporter activity, thus influencing the effectiveness of chemotherapy. These results highlight the importance of understanding diet–drug interactions in cancer therapy to improve treatment outcomes and reduce side effects. In conclusion, dietary modifications and supplements should be carefully considered in cancer treatment plans to optimize therapeutic efficacy.

## 1. Introduction

The effectiveness and safety of anticancer therapy can be significantly influenced by different active compounds not only included in the daily diet but also in dietary supplements, which play crucial roles in modulating the activity of multidrug resistance (MDR) transporters. These transporters, such as multidrug resistance protein 2 (MRP2), breast cancer resistance protein (BCRP), and P-glycoprotein (P-gp), are pivotal in mediating drug efflux from cancer cells, thus contributing to the development of chemotherapy resistance [1]. Dietary components and supplements can either inhibit or induce these transporters, impacting drug bioavailability and therapeutic outcomes. The inhibition can lead to increased intracellular concentrations of chemotherapeutic agents, enhancing their cytotoxic effects on cancer cells [2,3]. Conversely, some dietary supplements may induce the expression of MDR transporters, potentially reducing the effectiveness of chemotherapy. Understanding the intricate interactions between diet, dietary supplements, and MDR transporters is crucial for optimizing cancer therapy. This knowledge can help in the development of dietary guidelines and supplement recommendations for patients undergoing chemotherapy, ensuring enhanced efficacy and reduced adverse effects [4,5]. 

The primary aim of this study is to investigate the impact of diet and dietary supplements on the function and expression of key MDR transporters, namely, MRP2, BCRP, and P-gp. Specifically, this study seeks to achieve the following: (i) elucidate the mechanisms: understand how various dietary components and supplements modulate the activity and expression of MRP2, BCRP, and P-gp in vitro and in vivo; (ii) evaluate the effects: assess the impact of these dietary factors on drug bioavailability, intracellular drug accumulation, and overall therapeutic outcomes in cancer treatment; and (iii) identify potential interactions: investigate potential beneficial or adverse interactions between dietary supplements and chemotherapeutic agents, with a focus on enhancing drug efficacy while minimizing toxicity.

## 2. Methods

A review of publications was conducted based on the PubMed and Google Scholar databases, together with reference lists of all chosen articles. The keywords used to search for publications were combinations of the following words: “food–drug interactions”, “inhibition”, “induction”, “natural compounds”, “drug transporters”, “P-gp”, “MRP2”, “BCRP”, “multidrug resistance”, “cancer”. The selection was limited to in vitro and in vivo studies using anticancer drugs and natural substances found in the daily diet, which could reverse multidrug resistance in cancer. Particular attention was given to studies describing the structure–activity relationship. A time limit covering the period 2017–2024 was introduced. No language constraints were introduced. All figures were made using ChemDraw 22.2.0 software.

## 3. Function of Selected Carriers Involved in Transport of Anticancer Drugs

### 3.1. Multidrug Resistance-Associated Protein 2—MRP2

**Figure 1:**
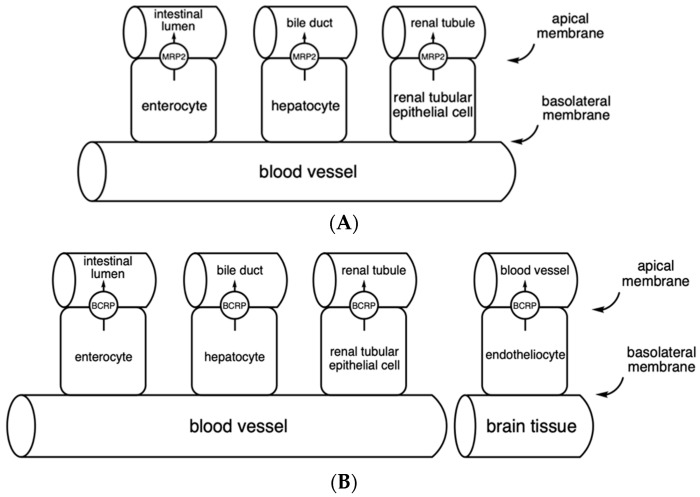
Localization of the MRP2 (**A**) and BCRP (**B**) transporters and the transport direction of their substrates in cells of the intestine, liver, kidney (**A**,**B**), and blood–brain barrier cells (**B**) according to [7,9].

Multidrug resistance-associated protein 2 (MRP2) is a member of the C subfamily of the superfamily of ATP-binding cassette (ABC), encoded by the *ABCC2* gene. It consists of 1545 amino acids forming three transmembrane domains and two ATP-binding domains. MRP2 is mostly expressed in the apical membrane of the tubular epithelial cells of the liver, kidney, and small intestine (Figure 1A). It is one of the efflux transporters and its main role is to pump out endogenous and exogenous substances from the cells. Its expression in the intestine limits the absorption of xenobiotics from the gastrointestinal tract, while in the liver, it facilitates the elimination of positively charged drugs and bilirubin glucuronate into the bile. It reduces the bioavailability of the drugs that are its substrates, including methotrexate, cisplatin, irinotecan, doxorubicin, ceftriaxone, ampicillin, and saquinavir. Its expression is increased in tumor tissues (including lung cancer, liver cancer, gastric cancer, squamous cell carcinoma of head and neck, and ovarian cancer), where it contributes to multidrug resistance by reducing intracellular drug accumulation [6,7].

In recent years, in vitro and in vivo studies have been conducted to find dietary-derived compounds that can affect the activity of the MRP2 transporter and help overcome multidrug resistance in cancer, what is discussed below. 

### 3.2. Breast Cancer Resistance Protein—BCRP

Breast cancer resistance protein (BCRP) belongs to the ABC superfamily of transporters. It is encoded by the *ABCG2* gene and consists of 655 amino acids forming six α-helices. In the human body, it is localized on the apical side of enterocytes, hepatocytes, renal proximal tubule cells, and blood–brain barrier endothelial cells (Figure 1B). As an efflux transporter, it plays an important role in protecting cells from toxic compounds. In cancer cells, it is responsible for pumping drugs outside the cell, thereby contributing to multidrug resistance. Substrates of this transporter are endogenous compounds including urea, estrone-3-sulfate, and dehydroepiandrosterone sulfate and drugs including rosuvastatin, topotecan, sunitinib, grepafloxacin, acyclovir, cimetidine, methotrexate, and sulfasalazine [8,9].

### 3.3. P-Glycoprotein—P-gp

One of the best-studied and described xenobiotic transporters is P-glycoprotein (P-gp, ABCB1, MDR1), a member of the ABC family. P-gp is a glycosylated and phosphorylated protein composed of 1280 amino acids of 170 kDa occurring in several isoforms. It consists of two units, each containing an intracellular hydrophilic nucleotide-binding domain (NBD1 and NBD2) and a transmembrane domain (TMD1 and TMD2) composed of six hydrophobic α-helices (TM1-12). Within the NBD domain, ATP is hydrolyzed to ADP and Pi, used to translocate molecules across the cell membrane, while the TMD domains are responsible for substrate recognition and transport pathway determination [10,11,12]. P-gp is thought to have at least three substrate-binding sites and one allosteric regulatory site [10]. It is a transporter for many substrates that differ in molecular weight, structure, and function. It transports both low-mass molecules, such as amino acids, carbohydrates, and organic cations, and macromolecules, including proteins and polysaccharides [10,12]. Most substrates are lipid-soluble and have slightly amphipathic and hydrophobic properties. Often, substrates contain a positively charged nitrogen atom and aromatic rings [12]. P-gp substrates include digoxin, fexofenadine, loperamide, quinidine, or vinblastine [13], and they are localized in many tissues; of particular importance are the barrier tissues. Its presence has been found in the endothelium of cerebral vessels, kidneys, liver, lungs, ovaries, stomach, enterocytes of intestinal villi, and intestinal mucosa, as well as in the vascular endothelium of cancerous tumors [11]. P-gp is an efflux transporter and thus has a primarily protective function of shedding endogenous and exogenous toxic compounds outside the cell, thereby reducing the availability of xenobiotics. It also influences the development of multidrug resistance in antineoplastic, anticancer, and antiepileptic drug therapies. Its overexpression is associated with drug-resistant epilepsy and cancers of the hematopoietic system, brain, intestine, and kidney [11,12].

## 4. Influence of Polyphenolic Compounds on MDR Transporters

### 4.1. Catechins

Catechins are flavan derivatives, predominantly found in green tea (*Camellia sinensis* L. (Kuntze)). The most abundant green tea catechin is epigallocatechin gallate (EGCG), known for its antioxidant properties. Green tea is widely consumed both as a beverage and in dietary supplement form, where it is marketed for its potential benefits in supporting cardiovascular health, weight management, and antioxidant protection. The high concentration of catechins in green tea makes it a popular choice for people seeking natural antioxidant supplements [14]. Additionally, cocoa and dark chocolate are rich sources of catechins, particularly epicatechin (EC). These compounds contribute to the antioxidant capacity of cocoa products and are linked to various health benefits, including improved cardiovascular function and reduced risk of chronic diseases [15].

Some studies have highlighted the role of stereochemistry of green tea catechins in regulating the efflux transport rather than the absorption transport in the Caco-2 monolayers. Better transcellular permeability in the efflux transport was shown by trans catechins when compared to the corresponding cis (epi) catechins. In addition, after incubation with catechins, a significant increase in the expression of MRP2 and BCRP and decrease in the expression of P-gp in Caco-2 cells was observed [16]. Another study showed the combined effect of catechins and other phytochemicals present in green tea, namely, caffeine and theanine but also serine and glycine, on Caco-2 monolayers. The cells were incubated with EGCG, epicatechin gallate (ECG), or EC, which are substrates for MRP2, combined with caffeine, theanine, serine, or glycine. The MRP2 expression in the cells treated with EGCG and EC was increased 1.58- and 2.98-fold, respectively, while no significant changes were seen for ECG. The addition of caffeine, theanine, serine, and glycine caused a decrease in MRP2 expression, increased by EGCG. Only glycine caused a decrease in EC-enhanced MRP2 expression. The results of the study suggest that caffeine, theanine, glycine, and serine in tea may increase catechin transport by decreasing the expression of the catechin-enhanced efflux transporter MRP2 [17]. 

A study on Caco-2 cells was performed with BCRP substrate H33342 and BCRP inhibitor Ko143 with sodium fluoride. The cells pretreated with a green tea infusion, ECG, and EGCG, showed a significant increase in intracellular accumulation of the substrate, while the increase for EC and epigallocatechin (EGC) was not statistically significant. An analogous study was also conducted using aflatoxin B1 as a substrate of BCRP. The ejection rate of the aflatoxin B1 after preincubation with a green tea infusion and ECG, EGC, and EGCG was significantly reduced. The results suggest an inhibitory effect of the catechins present in green tea on the activity of the BCRP transporter [18]. 

The effect of EGCG on pharmacokinetics of tacrolimus and cyclosporine A was investigated in an in vivo study. The compounds were administered to rats (n = 42), with or without EGCG. Coadministration of EGCG caused a reduction in the C_max_ and AUC of tacrolimus, and distribution/elimination profiles were enhanced. The C_max_ and AUC of cyclosporine A were increased by ECGC at a dose of 3–30 mg/kg but decreased at a dose of 100 mg/kg. To investigate the underlying mechanism, the rats were administered EGCG (3, 10, 30, and 100 mg/kg for 7 days) or CYP3A (ketoconazole, 75 mg/kg) or P-gp (verapamil, 24 mg/kg) inhibitors. The mRNA levels of the drug-metabolizing enzymes, drug transporters (including MRP2), and nuclear receptors were then examined. Compared to the control, untreated group, a reduction in the amount of mRNA of MRP2 was observed in liver cells by 21.13, 47, 60, 40.41, and 25.22%, respectively. In the intestinal cells, a reduction in the amount of mRNA of MRP2 was also noted by 13.89, 47.77, 36.11, and 13.50%, respectively. This suggests an inhibitory effect of EGCG on MRP2 expression [19].

### 4.2. Quercetin and Its Derivatives

Quercetin is one of the most widespread plant flavonoids. It is found in vegetables such as onions and tomatoes, in fruits such as berries, apples, and grapes, and medicinal plants such as ginkgo biloba and St. John’s wort. Quercetin and its derivatives are available in numerous dietary supplements designed to enhance immune function and reduce oxidative stress. These supplements often combine quercetin with vitamin C or bromelain to improve absorption and efficacy [20,21]. Quercetin exhibits many health-promoting properties, such as cardioprotective, anti-inflammatory, antioxidant, and antianaphylactic, and these make it a popular and widely available dietary supplement. These products typically contain 200–1500 mg of quercetin aglycone per tablet or capsule, and the manufacturers’ recommended daily dose is 150–4000 mg. Compared to supplementation, the average daily intake of quercetin from food is 5–40 mg, which is up to 100 times lower than the value recommended by manufacturers [22,23,24]. 

Mohos V. et al. [22] investigated the effects of quercetin and its metabolites, quercetin 3′-sulfate, quercetin 3-glucuronide, isorhamnetin, and isorhamnetin 3-glucuronide, on the activity of the MRP2 transporter in an inverted insect cell membrane vesicles model, and CDCF was used as the substrate for MRP2. A statistically significant decrease in the transport of CDCF by MRP2 was observed when each flavonoid was administered at a concentration of 20 μM. Isorhamnetin 3-glucuronide, quercetin 3′-sulfate, and quercetin 3-glucuronide showed stronger impacts on the MRP2 activity (IC_50_ = 14.9, 19.6, and 24.2 μM, respectively) than hydrophobic compounds—quercetin and isorhamnetin (IC_50_ > 30 μM). In the same study, the effect of these flavonoids on the activity of BCRP was also examined. A statistically significant decrease in the transport of a specific fluorescent substrate (lucifer yellow) was induced by 0.05 μM of quercetin, 0.2 μM of quercetin-3′-sulfate, 1.0 μM of quercetin-3-glucuronide, 0.05 μM of isorhamnetin, and 20 μM of isorhamnetin-3-glucuronide. Quercetin and isorhamnetin were potent inhibitors of BCRP (IC_50_ = 0.13 and 0.06 μM, respectively); quercetin-3′-sulfate also showed strong inhibition (IC_50_ = 3.20 μM), whereas the glucuronides quercetin-3-glucuronide and isorhamnetin-3-glucuronide were weak inhibitors (IC_50_ = 13.5 μM, IC_50_ > 30 μM, respectively).

MDCKII-MRP2 is a specialized cell line derived from Madin-Darby canine kidney (MDCK) cells, which is a widely used model for studying epithelial cell functions. This specific cell line has been genetically engineered to overexpress the human MRP2. The cells were treated with phenolsulfonphtalein (MRP2 substrate), probenecid (MRP2 inhibitor), or 5 or 10 μM quercetin. Quercetin showed a statistically significant inhibitory effect on the MRP2 transporter at the higher concentration tested. The human colon adenocarcinoma cells (LS174T) were treated with vincristine (an MRP2 inducer) or quercetin. Quercetin at a concentration of 50 μM increased MRP2 mRNA expression three-fold compared to the control group and showed a stronger activating effect than vincristine [23]. 

In the in vivo study, the rats (n = 18) received the following: phosphate buffer (control group) or ketoconazole (CYP3A4 inhibitor) or verapamil (P-gp inhibitor) or a low (2.5 mg/kg), medium (5 mg/kg), or high (10 mg/kg) dose of quercetin 3-O-β-D-glucoside for 7 days. The amount of MRP2 transporter mRNA was significantly reduced by quercetin 3-O-β-D-glucoside at low, medium, and high doses by 66.03, 34.53, and 28.66% in the small intestine, respectively, and in the liver by 68.86, 32.70, and 20.44%, respectively, compared to the control group. In the groups with low, middle, and high doses of quercetin 3-O-β-D-glucoside, the amount of BCRP mRNA was reduced by 63.83, 27.52, and 43.68%, respectively, in the small intestine. However, in the liver, the middle- and high-dose quercetin 3-O-β-D-glucoside groups decreased the BCRP mRNA expression levels by 29.23 and 29.23%, respectively, and only the low-dose quercetin 3-O-β-D-glucoside group showed a significant reduction (56.15%). The effect was not dose-dependent. A low dose of quercetin 3-O-β-D-glucoside showed the strongest inhibitory effect on the MRP2 and BCRP transporter. Similar results were obtained when MRP2 and BCRP protein expression was examined. Quercetin 3-O-β-D-glucoside at low, medium, and high doses reduced MRP2 protein expression by 75.95, 42.60, and 50.84%, respectively, in the intestine compared to the control group, while in the liver, only the low dose of the substance showed a statistically significant 70.70% reduction in expression. In the low-, middle-, and high-dose quercetin 3-O-β-D-glucoside groups, BCRP protein expression was decreased by 70.61, 32.93, and 36.44%, respectively, in the small intestine and by 69.78, 27.73, and 30.86%, respectively, in the liver. The effect was also not dose-dependent [24]. 

Oral administration of quercetin (50, 100, and 250 mg/kg) to rats for 7 days had no effect on mRNA expression in the liver and kidney, while in the intestine, after administration of 100 mg/kg quercetin, mRNA expression was 15.4 times higher compared to the control group. The difference in MRP2 mRNA expression in different organs may have been due to higher concentrations of quercetin in the intestine than in the liver and kidney as a result of the first-pass effect. A further study using phenolsulfonphthalein and orally administered quercetin once or for 7 days at doses of 50, 100, and 250 mg/kg showed no significant changes in the pharmacokinetics of phenolsulfonphthalein [23]. In a recent in vivo experiment, the control group was orally administered docetaxel, and the study group was orally administered quercetin (100 mg/kg) and docetaxel. A significant increase in AUCinf was observed in the study group from 141.35 ± 35 to 251 ± 65, suggesting an inhibitory effect of quercetin on MRP2 transporter activity. The concentration of quercetin in the intestine was 18.1 mM, which may be a concentration higher than the IC_50_ of P-gp and CYP3A4 for quercetin. This suggests that the increased AUCinf may also have been due to the inhibition of P-gp and CYP3A4 activity. The total plasma concentration of quercetin was 78.1 μM, but its free plasma concentration was predicted to be much lower than the IC_50_ value against MRP2 (higher than 50 μM). It is caused by quercetin binding to plasma protein extensively. This might be the reason for incoherent results between in vitro and in vivo studies. The authors suggest that quercetin modulates the function and expression of MRP2 in vitro, while under in vivo conditions, there is little likelihood of the interaction [23].

### 4.3. Apigenin

Apigenin is a flavonoid predominantly found in parsley, celery, and chamomile tea. It is also present in significant amounts in fruits like oranges and certain herbs such as thyme and oregano. Apigenin is widely studied for its potential health benefits, including anti-inflammatory, antioxidant, and anticancer effects. Dietary supplements containing apigenin are marketed for their potential to support immune function, reduce inflammation, and promote relaxation and sleep [25].

The inhibitory potential of apigenin was investigated in a sole study. Oral administration of dasatinib after seven days’ pretreatment of apigenin (40 mg/kg) significantly increased the plasma concentration of dasatinib compared to dasatinib alone. The significant inhibition of the BCRP protein (66.77% inhibition in hepatic cells and 41.06% inhibition in intestinal cells) was noted in the apigenin-pretreated rats compared to dasatinib alone. The bioavailability of dasatinib was enhanced by the significant inhibition of CYP3A2, P-gp, and BCRP1 expression and the suppression of its hepatic and intestinal metabolism [26].

### 4.4. Licochalcone A

Licochalcone A is a chalconoid predominantly found in the root of different licorice species. This compound is noted for its anti-inflammatory, antioxidant, and antimicrobial properties. It is used in various traditional medicines and is also being studied for its potential therapeutic applications in modern medicine. Licochalcone A is present in dietary supplements aimed at promoting skin health and reducing inflammation. These supplements often source licochalcone A from licorice root extracts, taking advantage of its potential benefits in managing skin conditions and enhancing overall wellness [27,28].

The effect of licochalcone A on the efflux transporters in the multidrug resistance cancer cells was examined using R482-HEK293 cells—a genetically engineered cell line derived from the human embryonic kidney 293 (HEK293) cells, overexpressing the R482 variant of the human BCRP. The cells were treated with different doses of mitoxantrone or topotecan and with nontoxic concentrations of licochalcone A (0.5, 1, 2, and 3 μM). Licochalcone A significantly restored the chemosensitivity of the cells to mitoxantrone and topotecan in a concentration-dependent manner (mitoxantrone: IC_50_ = 18.75, 7.09, 7.83, and 6.22 nM, respectively; topotecan: IC_50_ = 90.05, 51.47, 44.11, and 42.41 nM, respectively). It also significantly reversed BCRP-mediated mitoxantrone and topotecan resistance in a concentration-dependent manner in S1-M1-80 (mitoxantrone: IC_50_ = 39.47, 6.80, 2.63, and 1.53 μM, respectively; topotecan: IC_50_ = 1.03, 0.61, 0.41, and 0.31 μM) and H460-MX20 cell lines (mitoxantrone: IC_50_ = 441.96, 86.46, 74.77, and 69.86 nM, respectively; topotecan: IC_50_ = 366.91, 143.89, 147.56, and 130.38 nM, respectively) [29].

### 4.5. Miscellaneous Flavonoids

Some studies have also focused on flavonoids, which are commonly found in plants used for their beneficial effect on human health. These included ginkgo and St. John’s wort (e.g., amentoflavone, apigenin, sciadopitysin), red clover (biochanin A), Chinese skullcap (e.g., oroxylin A), citrus fruits (e.g., diosmin, naringenin, isosinensetin, tangeretin, and sinensetin), kale, spinach, and broccoli (e.g., kaempferol, kaempferide) or propolis (e.g., chrysin, genkwanin). These compounds are incorporated into dietary supplements for their antioxidant, anti-inflammatory, and potential therapeutic properties and for metabolic health [30,31,32,33]. 

The effect of almost 100 flavonoids on BCRP transporter activity was investigated using mitoxantrone as a substrate. The flavonoids at 50 μM or the highest nontoxic concentration applied to MDCKII-BCRP cells showed significant, over 50%, inhibition of BCRP transporter activity. Next, the concentration–inhibitory effect relationship of the flavonoids was studied. The strongest inhibitor was amentoflavone (IC_50_ = 4 ± 1 μM), followed by kaempferide (IC_50_ = 5 ± 1 μM), kaempferol (IC_50_ = 15 ± 2 μM), diosmin (IC_50_ = 17 ± 3 μM), naringenin (IC_50_ = 19 ± 2 μM), chrysin (IC_50_ = 20 ± 3 μM), apigenin (IC_50_ = 24 ± 3 μM), biochanin A (IC_50_ = 24 ± 4 μM), licochalcone A (IC_50_ = 33 ± 8 μM), and genkwanin (IC_50_ = 37 ± 5 μM). The study was further conducted on rats (n = 39), which received a single dose of amentoflavone, licochalcone A, diosmin, chrysin, or naringenin at 30 mg/kg; genkwanin at 10 mg/kg; apigenin, biochanin A, kaempferol, or kaempferide at 25 mg/kg; or Ko143 at 25 mg/kg (positive control group), followed by the administration of mitoxantrone after 30 min. A 25.62% increase in the AUC0-t value of mitoxantrone was observed in the positive control group. After the administration of apigenin, naringenin, licochalcone A, kaempferol, and chrysin, an increase in AUC0-t values was observed by 30.10–81.97%, which was higher than that of the positive control group. The results indicate a significant inhibitory effect on the BCRP transporter activity of 11 of the 99 flavonoids tested [34]. A similar in vivo study on naringenin was also performed in rats (n = 12), which were provided with a single oral dose of dasatinib, with or without naringenin pretreatment (150 mg/kg daily for 7 days). The plasma mean concentration of dasatinib was significantly enhanced in the pretreated group compared with the non-pretreated animals [35]. 

A total of 75 flavonoids were examined towards their inhibitory effect on P-gp using MDR1–MDCKII cells. The concentration range of flavonoids screened was 0–100 μM except for tangeretin, isosinensetin, sciadopitysin, and oroxylin A, which were screened at 0–150 μM for further IC_50_ assay. Isosinensetin, tangeretin, sinensetin, sciadopitysin, and oroxylin A showed significant inhibition with the following IC_50_ values: 4.2, 12.66, 18.9, 53.42, and 78.33 μM, respectively. The wild cancer cells MX-1 and taxol-resistant MX-1/T cells were used to investigate the effect of P-gp inhibition by flavonoids (isosinensetin 8.4 μM, tangeretin 25.3 μM, sinensetin 37.8 μM, sciadopitysin 106.8 μM, oroxylin A 155.6 μM) on taxol (75μM) cytotoxicity. All flavonoids mentioned before and verapamil increased taxol toxicity in both cell lines, and tangeretin, oroxylin A, and sciadopitysin showed a much stronger reduction in cell viability than verapamil. Tangeretin and sciadopitysin significantly augmented taxol cytotoxicity in MX-1/T cells compared with MX-1 cells, and these might be used to reverse multidrug resistance in cancer [36].

An interesting study compared the activity of a number of polyphenols present in sour cherry (*Prunus cerasus*) fruits. The compounds were tested for their impact on the P-gp activity on the NIH 3T3 mouse fibroblast cell line and its human P-gp-overexpressing analogue, NIH 3T3 MDR1. The results showed that quercetin, quercetin-3-glucoside, narcissoside, and ellagic acid lowered the ATPase activity of P-gp and increased the accumulation of calcein and daunorubicin by P-gp-positive cells. Cyanidin-3O-sophoroside, catechin, naringenin, kuromanin, and caffeic acid also augmented the ATPase activity of P-gp, but they had a weaker impact on the intracellular accumulation of calcein and daunorubicin. Polyphenols such as epicatechin, trans-ferulic acid, oenin, malvin, and chlorogenic acid presented no effect. What is interesting is that two stereoisomers, catechin and epicatechin, showed different effects. The application of quercetin, naringenin, or ellagic acid with verapamil, a P-gp inhibitor, led to an additive or synergistic inhibitory effect that might be used in further studies to reverse multidrug resistance [37].

### 4.6. Sinapic Acid

Sinapic acid is a phenolic compound found in various fruits, vegetables, and grains. It is particularly abundant in canola (rapeseed) oil, mustard seeds, and certain berries like blueberries and cranberries. Sinapic acid is known for its antioxidant and anti-inflammatory properties, and it is increasingly incorporated into dietary supplements aimed at promoting cardiovascular health and reducing oxidative stress [38].

A sole study examined the impact of sinapic acid on BCRP protein. A single dose of dasatinib was given to rats (n = 6), with or without sinapic acid pretreatment (20 mg/kg per day for 7 days). A significant inhibition of the BCRP protein (51.44% inhibition in hepatic cells and 50.48% inhibition in intestinal cells) was observed in the pretreated rats compared with the animals given dasatinib alone. An increase in the bioavailability of dasatinib was found via modulation of CYP3A2, P-gp, and BCPR protein expression [39]. 

### 4.7. Resveratrol 

Resveratrol is a stilbenoid found in a variety of foods, most notably in the peels of red grapes, translating to its presence in red wine and contributing to its potential health benefits. Additionally, resveratrol can be sourced from peanuts, dark chocolate, and certain types of Japanese knotweed (*Polygonum cuspidatum*), which is often used in supplements [40,41].

The effect of resveratrol on the expression of genes associated with multidrug resistance and the proteins encoded by these genes, including the *ABCB1* gene encoding P-gp, was investigated. Human gastric cancer cell lines were used for the study: two daunorubicin-resistant (EPG85-257RDB; RDB) and mitoxantrone-resistant (EPG85-257RNOV; RNOV) and one cytostatic-sensitive (EPG85-257P, a control cell line). Cells were treated with 30 or 50 μM resveratrol for 72 h. Resveratrol at both concentrations showed a statistically significant reduction in the expression of the *ABCB1* gene, among others. In the RDB cell line, a reduction in the expression of all the genes tested and selected proteins, including P-gp, was observed. Based on the results, it can be concluded that resveratrol, after long exposure, can reduce multidrug resistance in cancer cells by decreasing gene and protein expression [42]. Another study was conducted to evaluate the effects of resveratrol on the expression and function of P-gp on Caco-2 in CEM/ADR5000 cell lines. Resveratrol at concentrations of 10–100 μM inhibited P-gp efflux function by causing the accumulation of rhodamine 123 with calcein in a dose-dependent manner. In the rhodamine 123 assay, the potency of P-gp inhibition by resveratrol was 2.11–3.90 times higher than that caused by verapamil, which served as a positive control. In a calcein assay, the inhibitory potency was 1.64–4.6 times stronger than that caused by verapamil. Administration of 20 μM resveratrol also resulted in a significant increase in the cytotoxicity of doxorubicin, allowing the dose of the drug to be reduced while maintaining the effect. The cytotoxicity of doxorubicin combined with resveratrol was IC_50_ = 1.23 μM, compared to IC_50_ = 4.15 μM for doxorubicin alone. A 48 h observation of the Caco-2 cell line also noted significantly lower P-gp mRNA levels due to the presence of 20 μM resveratrol. The results of the study confirmed the inhibitory effect of resveratrol on the efflux activity of ABC transporters, including P-gp, as well as their expression [43].

### 4.8. Curcumin 

Curcumin is a bioactive compound predominantly found in turmeric, a spice derived from the root of the *Curcuma longa* plant. It is widely used in traditional Indian and Southeast Asian cuisines and is the primary ingredient in curry powder, contributing to its distinctive yellow color and potential health benefits, such as anti-inflammatory and antioxidant effects [44,45].

An in vitro study was performed to examine how natural compounds can control BCRP expression in mixed conditions. Human oral squamous carcinoma OECM1 and head/neck cancer SASL90d cell lines were used in the experiment. Curcumin at the doses of 5, 10, and 15 μM and EGCG at the doses of 20, 30, and 50 μM significantly decreased BCRP levels and BCRP protein expression compared with the control group. The therapeutic effect of the tested compounds was also examined. Protoporphyrin IX accumulation was enhanced in EGCG- and curcumin-treated cells in a dose-dependent manner and was inversely correlated with the cell viability [46].

The study conducted on Caco-2, CEM/ADR5000, and CCRF-CEM cell lines was to test whether the combination of polyphenols, including curcumin, with doxorubicin showed synergistic effects in anticancer treatment. The results showed a beneficial effect of combining polyphenols with doxorubicin on the sensitization of cancer cells. The ability of the polyphenols used to inhibit P-gp activity was also investigated. The evaluation was based on the concentration of doxorubicin inside the cell. All polyphenols showed an increase in doxorubicin concentration. The results of the study presented evidence of curcumin’s inhibitory effect on P-gp in the Caco-2 and CEM/ADR5000 cell lines. A study on the CCRF-CEM cell line showing low expression of ABC transporters confirmed the absence of P-gp transporter activity [47]. The ability of curcumin to reverse multidrug resistance was also investigated on doxorubicin-resistant colon cancer cells of the SW620/Ad300 line. Curcumin administration showed an increase in doxorubicin-induced cytotoxicity and cell apoptosis, which was due to the reversal of resistance mediated by P-gp action. Further studies indicated an increase in doxorubicin accumulation in resistant cells after curcumin administration due to its inhibitory effect on P-gp activity. No changes were observed in the doxorubicin-sensitive SW620 cell line. The above data suggest the ability of curcumin to reverse multidrug resistance during anticancer treatment [48].

## 5. Effect of Terpenoids and Sterols on MDR Transporters

Menthol, geraniol, caryophyllene, and carnosol are naturally occurring terpenoid compounds with notable health benefits. Menthol is a monoterpene primarily found in the essential oils of different mint plants, such as peppermint (*Mentha × piperita*), and is widely used in foods, beverages, and topical products for its cooling and soothing effects. Geraniol, a monoterpene alcohol, is abundant in essential oils of roses, geraniums, and citronella and contributes to their fragrance. Caryophyllene, a sesquiterpene, is present in black pepper, cloves, and cinnamon, known for its spicy aroma and potential anti-inflammatory properties. Carnosol is a diterpenoid, found in rosemary (*Rosmarinus officinalis*) and sage (*Salvia officinalis*) and is noted for its antioxidant and cytotoxic activities [49,50].

### 5.1. Menthol

An in vitro study on HepG2 cells was conducted to evaluate the effect of menthol on the expression of MRP2 and its impact on the cytotoxicity of epirubicin and cisplatin. Exposure of the cells to menthol at concentrations of 50 and 100 μM increased the expression of MRP2 mRNA and led to a decrease in the intracellular accumulation of epirubicin and in the intracellular concentration of the epirubicin remaining. Menthol at a concentration of 10 μM had no significant effect on the expression of MRP2. Treatment with MK-571 (MRP2 inhibitor), but not verapamil (P-gp inhibitor), significantly attenuated the reduced intracellular accumulation of epirubicin. Both epirubicin and cisplatin had cytotoxic effects on HepG2 cells, but the decline in cell viability was significantly suppressed by 24 h exposure to menthol. These findings show that menthol leads hepatocellular carcinoma to develop resistance to anticancer treatments, such as epirubicin and cisplatin, through the induction of MRP2 [51].

### 5.2. Geraniol 

The potential effect of geraniol supplementation on restoring MRP2 activity inhibited by fructose as an inductor of metabolic syndrome was examined. Wistar rats were used to investigate the effects of geraniol on metabolic syndrome (MetS)-like conditions [52]. The animals were fed a standard commercial diet and received plain tap water (control group) or tap water with 10% fructose (FRU group) for 21 days to induce MetS-like conditions. In the geraniol treatment protocol, both control and MetS rats were administered either Tween 80 (control) or geraniol in Tween 80 (250 mg/kg/day). Supplementation with this natural compound restored MRP2 activity in fructose-fed rats [14].

### 5.3. β-Caryophyllene Oxide

Interesting in vitro studies were performed to investigate the ability of β-caryophyllene oxide (CRYO) at nontoxic doses to suppress efflux transporters and augment the response of hepatocellular carcinoma cells to sorafenib. Flow cytometry of fluorescent substrates’ export revealed that CRYO inhibited the efflux of rhodamine 123 (MDR1) and calcein (MRP1 and MRP2) but did not inhibit fluorescein (MRP3, MRP4, MRP5). Treatment of human liver Alexander cell sublines, both WT (wild-type) and R (with enhanced multidrug resistance), with sorafenib caused cell death, which was more marked in WT cells than in R cells. The IC_50_ value was lower in WT cells (1.2 ± 0.4 µM) than in R cells (3.3 ± 0.3 µM). An in vivo study in mice showed that CRYO inhibited sorafenib efflux, favored its intracellular accumulation, and enhanced its cytotoxic response [53]. In the in vivo study, Hepa1-6 wild-type (WT) and Hepa1-6/R (resistant) liver cancer cells were implanted in mice to evaluate the efficacy of sorafenib and its combination with CRYO. After 28 days, tumors developed with a final volume (FTV) of 7.9 ± 0.4 cm^3^ in the WT group and 6.6 ± 0.3 cm^3^ in the resistant group. Treatment with sorafenib alone reduced the tumor volume in WT cells by 38% (to 4.9 ± 0.5 cm^3^) and was less effective in resistant cells, showing a 20% reduction (to 5.3 ± 0.6 cm^3^), which was not statistically significant. However, the combination of sorafenib with CRYO significantly enhanced the treatment’s efficacy, reducing tumor volumes by approximately 65% and 58% in WT and in the resistant cells, respectively. Tumor weights at the study’s conclusion corresponded with these volume measurements. Additionally, high-performance liquid chromatography–mass spectrometry (HPLC-MS/MS) analysis revealed that coadministration of CRYO increased the intratumoral concentration of sorafenib. In WT tumors, sorafenib levels increased from 67 ± 6 nmol/g tissue to 87 ± 5 nmol/g with CRYO. In resistant tumors, sorafenib accumulation was initially lower (31 ± 3 nmol/g) but increased significantly (to 94 ± 5 nmol/g) with the addition of CRYO, suggesting CRYO enhances sorafenib’s accumulation and efficacy in tumor tissue.

In a study on human cholangiocarcinoma EGI-1 and TFK-1 cell lines, the loading value of 25 µM mitoxantrone was higher when it was administered with the BCRP inhibitor fumitremorgin C compared to mitoxantrone alone. A similar effect was observed with mitoxantrone and CRYO. The compound significantly increased the cytotoxicity caused by cisplatin, mitoxantrone, sorafenib, and 7-ethyl-10-hydroxy-camptothecin in EGI-1 and TFK-1 cell lines. It showed little or no effect with gemcitabine, 5-fluorouracil, and oxaliplatin. A further in vivo study was performed in mice (n = 15) treated with 50 mg/kg CRYO, cisplatin, or a combination of both substances. A significant inhibitory effect on the BCRP transporter was observed only in the third group [54].

### 5.4. Carnosic Acid and Carnosol

Carnosic acid, carnosol, and rosemary extract (containing 23.2% carnosic acid and 12.4% carnosol) at concentrations up to 100 μg/mL were applied to a HepG2 cell line model for 24 h. Carnosic acid significantly increased the expression of the MRP2 transporter in contrast to rosemary extract and carnosol, which increased the expression only slightly. Increasing the expression of the MRP2 transporter may be one of the methods used in chemoprevention [55].

### 5.5. Beta-Sitosterol

Beta-sitosterol, a plant sterol with cholesterol-lowering properties, is naturally present in a variety of foods including nuts, seeds, and plant-based oils. Significant sources include peanuts, almonds, and avocados. It is also found in high amounts in wheat germ and soy products. These dietary sources contribute to its potential benefits in managing cholesterol levels and supporting heart health. In dietary supplements, beta-sitosterol is often derived from plant sources such as soybeans, corn, and pine trees. These supplements are commonly marketed for their benefits in reducing cholesterol levels and alleviating symptoms of benign prostatic hyperplasia (BPH). They provide a concentrated source of beta-sitosterol compared to typical dietary intake [56].

An in vitro study was conducted on HCT116/OXA cells, human colon cancer cells resistant to oxaliplatin. A significant decrease in the IC_50_ values of oxaliplatin was observed after administration of β-sitosterol at concentrations of 12.5, 25, and 50 μM, suggesting the sensitization of cells to its effects, with IC_50_ values of 222.74 ± 9.53, 98.29 ± 5.65, and 72.06 ± 4.52 μM, respectively, compared to 279.81 ± 11.69 μM in the control group. In further studies to determine which transporter associated with multidrug resistance is affected by β-sitosterol, the intracellular accumulations of mitoxantrone (BCRP substrate), rhodamine 123 (P-gp substrate), and CDCF (MRP2 substrate) were evaluated. β-sitosterol did not affect the intracellular accumulation of rhodamine 123 and CDCF but significantly dose-dependently increased the accumulation of mitoxantrone, indicating an inhibitory effect of β-sitosterol on BCRP transporter activity. BCRP expression in cells with oxaliplatin applied was not significantly reduced. The combination of oxaliplatin with 50 μM β-sitosterol showed significant inhibition of the expression of this transporter. The researchers also conducted an in vivo study on mice (n = 24) inoculated with HCT116/OXA cells to induce tumors and administered with β-sitosterol at 10 mg/kg, oxaliplatin, or the two substances combined. In the oxaliplatin group, a slight inhibition of tumor growth was observed. No changes were observed in the β-sitosterol-only group compared to the control group. Significant inhibition from day 12 of therapy was noted in mice given oxaliplatin combined with β-sitosterol, with a tumor volume 87% smaller compared to the control group. The results suggest that the combined use of oxaliplatin with β-sitosterol may have a beneficial effect on the treatment of colorectal cancer [57].

## 6. Effect of Alkaloids on MDR Transporters

### 6.1. Berberine

Berberine is an alkaloid predominantly found in several traditional medicinal plants, including Berberis species (such as *Berberis vulgaris*), *Coptis chinensis* (golden thread), and *Phellodendron amurense* (Amur cork tree). These plants are commonly used in traditional Chinese medicine for their antimicrobial, anti-inflammatory, and blood-sugar-lowering effects. Berberine is commonly present in dietary supplements recommended for hyperglycemia and atherosclerosis [58].

An in vitro study on the MCF-7/DOXFluc breast cancer cell line showed that berberine administered with doxorubicin in a 2:1 ratio optimally enhanced the antiproliferative effects of doxorubicin by increasing its concentration and retention time in tumor cells as a result of facilitating its uptake by inhibiting P-gp activity. The group treated with a combination of berberine (Ber) and doxorubicin (DOX) showed a smaller tumor volume compared to the other groups. While the group that received only Ber (10 mg/kg) or only DOX displayed a slight decrease in tumor growth compared to the control group, the combination treatment resulted in a significantly greater inhibition of tumor growth. Notably, the body weight of the nude mice in the DOX-only group significantly decreased, likely due to the toxicities and side effects associated with DOX. An in vivo study in mice using D-luciferin sodium indicated that berberine could significantly reduce P-gp activity. Berberine use caused the down-regulation of P-gp, as determined by Western blot and immunohistochemical tests. Based on the results of high-performance liquid chromatography, an increase in the distribution of doxorubicin into tumor tissues was observed after the administration of berberine. The above data suggest that berberine is an inhibitor of P-gp and causes a down-regulation of its expression [59]. Different results were observed by Yu et al. [60], who performed a study with berberine-rich *Coptidis rhizoma*. The rats were administered cyclosporine, a known substrate for P-gp, and were pretreated with a decoction of *Coptidis rhizoma* (1 g/2 mL/kg) 0.5 h before cyclosporine administration or the decoction twice a day, and the seventh dose was given 0.5 h before cyclosporine. The dose of *Coptidis rhizoma* corresponded to the average daily dose of 10 g used in clinical practice in humans. The results indicated that the administration of one and seven doses of *Coptidis rhizoma* decoction significantly reduced the C_max_ of cyclosporine by 56.9% and 70.4%, respectively, and the AUC0-t by 56.4% and 68.7%, respectively. A study on LS 180 human colon cancer cells showed a significant reduction in the intracellular accumulation of rhodamine 123 after administration of 2.5, 5.0, and 10 μM berberine. The results described indicate that P-gp is activated after oral ingestion of *Coptidis rhizoma* decoction.

### 6.2. Capsaicin 

Capsaicin is the active compound responsible for the spicy heat in chili peppers (*Capsicum* species). It is most abundantly found in varieties such as cayenne, jalapeño, and habanero peppers. Capsaicin contributes to the characteristic pungency of these peppers and is recognized for its potential health benefits, including analgesic, anti-inflammatory, and metabolic effects. In dietary supplements, capsaicin is commonly included in formulations designed to support weight loss, enhance metabolism, and relieve pain [61].

A sole study described the effect of capsaicin on MRP2 transporter. The rats (n = 36) were orally administered capsaicin (3.0 mg/kg), MRP2 transporter inhibitor—cyclosporine A, or olive oil as the control group. On the last day of treatment, all individuals were given vinblastine. The AUC0-t value of vinblastine in rats after capsaicin treatment was 1.3 times higher and 1.7 times higher after cyclosporine A treatment compared to the control group. A significant decrease in MRP2 expression (mRNA analysis) was also observed. The study suggests an inhibitory effect of capsaicin on the MRP2 transporter in rats [62]. 

### 6.3. Piperine

Piperine is an alkaloid found primarily in black (*Piper nigrum*) and long (*Piper longum*) peppers. It is well-known for its role in enhancing the bioavailability of various nutrients and drugs by inhibiting certain drug-metabolizing enzymes. Black pepper is the most common dietary source of piperine, and it is often included in dietary supplements aimed at improving nutrient absorption. Studies show that piperine can significantly increase the absorption of curcumin, a compound found in turmeric, which highlights its role in enhancing the effectiveness of dietary supplements [63]. 

The inhibitory effect of piperine on MRP2 transporter and/or BCRP transporter was evaluated in vivo. The rats (n = 16) received a single oral dose of silybin (50 mg/kg) or piperine (10 mg/kg) combined with silybin. The measured C_max_ values of silybin were 1.49 times higher for silybin A and 1.39 times higher for silybin B in the piperine-treated group. AUC0-t values were also higher by 146% and 181% for silybin A and silybin B, respectively. To investigate the mechanism by which the bioavailability of silybin was increased after piperine treatment, an in vitro study was conducted on models of the Caco-2, MDCKII-BCRP, and MDCKII-MRP2 cell lines. Caco-2 cells were incubated with silybin (10 μM) and piperine (up to 40 μM). The rate of silybin transport was higher in the direction from the basolateral side to the apical side than in the opposite direction and was 5.04 ± 0.48, respectively, for silybin A and 4.61 ± 0.39 for silybin B. A significant increase in transport from the apical to the basolateral side and a decrease in the silybin efflux ratio after piperine administration was observed. This suggests the inhibition of MRP2 and/or BCRP transporter activity by piperine in Caco-2 cells. In a study on the MDCKII-MRP2 cell line, a concentration-dependent, significant reduction in the efflux ratio of the CDCFDA compound (substrate for MRP2) was observed from 3.58 ± 0.52 to 1.40 ± 0.02 after piperine administration. This suggests that piperine is an inhibitor of the MRP2 transporter, which may have been the mechanism responsible for the increased bioavailability of silybin in the Caco-2 cells. Piperine concentration-dependently decreased the efflux ratio of silybin A from 2.34 ± 0.17 to 1.55 ± 0.38 and silybin B from 2.05 ± 0.22 to 1.32 ± 1.32 in the MDCKII-BCRP cell line. This suggests the inhibition of BCRP transporter activity by piperine, which may have been a mechanism for the increased bioavailability of silybin [64].

In the study by Kim et al. [65], the mice received piperine (10 mg/kg), capsaicin (5 mg/kg), and [6]-gingerol (5 mg/kg), a compound found in ginger. The substances were administered in two subsequent doses every 60 min (piperine) or every 30 min (capsaicin and [6]-gingerol), and then the animals received doxorubicin. None of the substances significantly affected the plasma concentration of doxorubicin. Based on the calculated tissue-to-plasma partition coefficients, it can be concluded that piperine significantly increased the level of distribution of doxorubicin to the kidney and liver, and capsaicin to the kidney, liver, and brain. [6]-gingerol did not affect the distribution of doxorubicin. The inhibition of P-gp may have caused a decrease in the secretion of doxorubicin into the urine and bile, which may have affected its accumulation in the previously mentioned organs.

## 7. Effect of Plant Juices on MDR Transporters

### 7.1. Rocket Juice

Diet–drug interactions with rocket (*Eruca vesicaria*) were explored. The rats (n = 64) were given the juice of fresh rocket leaves at doses of 1.0, 1.4, and 2.0 g/kg for 14 days. On the fifteenth day, half of each group was treated with cyclophosphamide or control saline solution. No changes in MRP2 protein expression were observed 24 h after cyclophosphamide administration. However, a significant increase was observed in MRP2 expression in liver cells after the administration of the highest dose of rocket leaf juice in both male and female rats. It should be considered that the consumption of rocket might negatively affect treatments with drugs whose pharmacokinetics depend on the action of ABC transporters and drugs that induce DNA damage [66].

### 7.2. Cranberry Juice 

An in vitro study examined the impact of cranberry juice on BCRP transporter, using the MDCKII-BCRP cell line model, mitoxantrone as a substrate, and Ko143 (a BCRP inhibitor) as a positive control group. Administration of cranberry juice at concentrations of 5.0, 2.5, and 1.3 mg/mL resulted in a decrease in the intracellular accumulation of mitoxantrone by 22, 17, and 15%, respectively. Quercetin, isorhamnetin, myricetin, cyanidin, protocatechuic acid, and scopoletin were detected in this juice, and cyanidin was the major constituent. Metabolites of these compounds, such as glucuronides and sulfates, showed an increase in intracellular accumulation of mitoxantrone by 19%. The results suggest that cranberry juice activates the BCRP transporter. The researchers also conducted an in vivo study examining the interaction between warfarin and cranberry juice. They found that consuming cranberry juice 0.5 h before taking racemic warfarin significantly lowered the maximum concentration (C_max_) and area under the curve (AUC_0-t_) for S-warfarin by 48% and 34%, respectively. Similarly, the C_max_ and AUC0-t for R-warfarin were reduced by 51% and 52%. Moreover, the area under the curve from time 0 to 10 h (AUC_0–10_) for both S- and R-warfarin decreased by 52% and 54%, respectively. In a different part of the study, administering cranberry juice 10 h after warfarin did not alter the C_max_ and AUC_0–t_ of either enantiomer. However, the half-life (t1/2) and the AUC from 48 to 96 h (AUC_48–96_) for S-warfarin increased significantly, by 267% and 126%, respectively. The results suggest that cranberry juice activated BCRP during absorption and inhibited the activity of this transporter during the elimination process [67].

Another study examined cranberry juice in rats. The animals (n = 12) were given gefitinib alone, or combined with cranberry juice (5 g/kg of cranberry as juice). Coadministration of gefitinib and cranberry juice caused a significant increase in C_max_ and AUC0-t values (28 and 55%, respectively). LS 180 cell line (intestinal human cancer adenocarcinoma) was used to evaluate the effect of cranberry juice on the activity of P-gp. Cranberry juice significantly reduced the intracellular accumulation of the P-gp substrate rhodamine 123 by 27%. The protein level of P-gp in rat enterocytes was decreased by 28%, and in hepatocytes it was increased by 39%. The protein levels of BCRP, CYP3A4, and CYP2D6 were decreased in both enterocytes (by 22, 24, and 38%, respectively) and hepatocytes (by 40, 25, and 30%, respectively). The authors suggest that the influence of cranberry juice on the absorption of gefitinib by modulating P-gp is negligible because of the opposite effects of cranberry juice on P-gp activity and its protein levels. Changes in the activities and protein levels of BCRP, CYP3A4, and CYP2D6 might explain the effect of cranberry juice on the pharmacokinetics of gefitinib [68].

## 8. Structure–Activity Relationship

**Figure 2:**
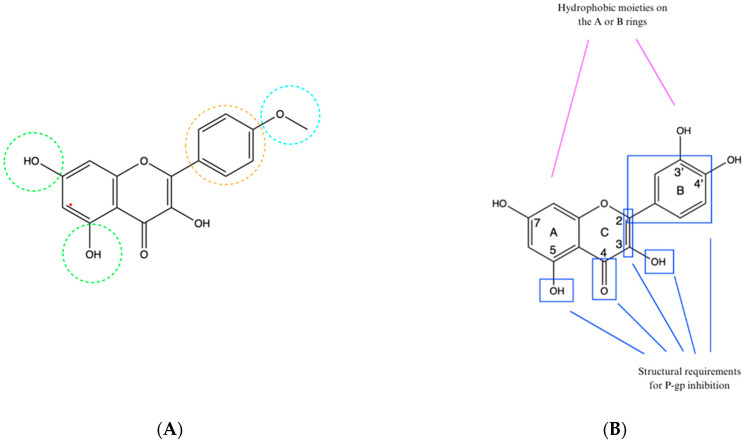

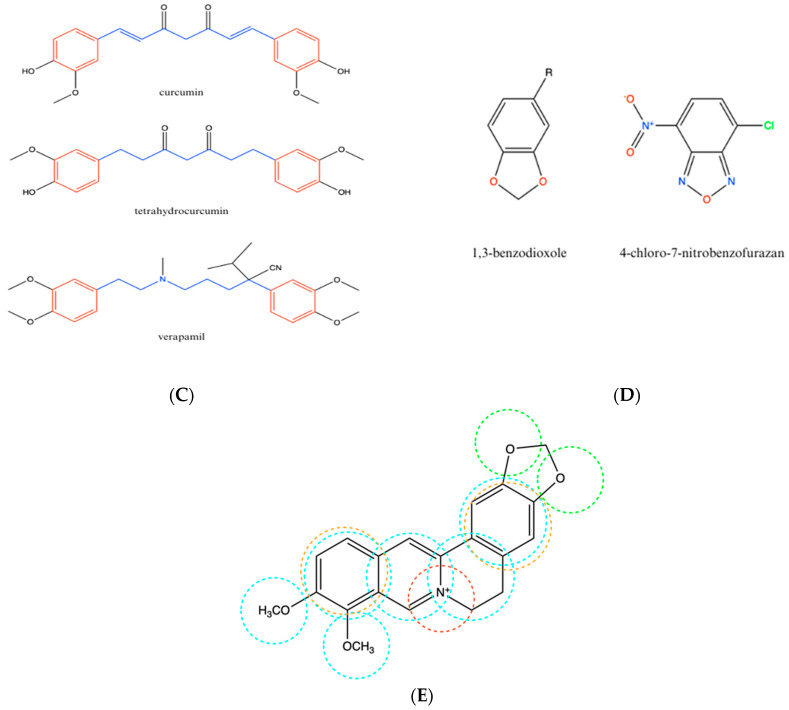
The structural requirements of natural compounds for inhibition of selected transporters. (**A**) The chemical structure of kaempferide (BCRP inhibitor), with the hydroxyl groups at positions 5 and 7, being hydrogen bond acceptors, in green, the aromatic ring B in orange, and the methoxyl hydrophobic group at the 4′ position in blue—prepared according to [34]. (**B**) Structural requirements in flavonoids for P-gp inhibition—prepared according to [69]. (**C**) Similarity of chemical structures of curcumin, tetrahydrocurcumin, and verapamil; the red color indicates the aromatic rings at the ends of the chains, and the blue color indicates the linker—prepared according to [72]. (**D**) Similarity of chemical structures of 1,3-benzodioxole and 4-chloro-7-nitrobenzofurazan—prepared according to [75]. (**E**) Structure of berberine with labeled structures responsible for bond formation. Aromatic rings are marked in orange, hydrophobic elements in blue, cations in red, hydrogen bond acceptors in green—prepared according to [78].

Despite the importance of the interaction between the natural compounds and different MDR transporters, only a few studies described the impact of the structural elements of the studied compounds on their activity towards the transporters. As far as BCRP transporter is concerned, a sole study was performed for flavonoids, based on the computational docking model. The results indicated that the presence of an aromatic ring, hydrophobic groups, and hydrogen bond acceptors was necessary for the inhibition of the BCRP transporter. Compounds containing in their structure an aromatic ring B (marked in orange in Figure 2A), a methoxyl group at the 4′ position (marked in blue in Figure 2A), hydroxyl and/or hydrophobic substituents at positions 5 and 7 (marked in green in Figure 2A) showed greater inhibitory activity. It was observed that the substitution at these positions with large substituents such as glucose could reduce the inhibitory effect or cause it to disappear [69]. Some more data exist on the effect observed towards P-gp activity, including the compounds representing different chemical classes discussed within this review. 

It is believed that flavonoids can interact with different sites on P-gp (substrate-binding site, other drug-binding sites, ATP-binding site, allosteric site, steroid-interacting region). In cellular studies, most flavonoids affected P-gp activity at concentrations of at least 10 μM but usually at higher values. Such levels are only achievable in the gut, probably after oral supplementation with these compounds. Flavonoid metabolites present in human plasma, due to their hydrophilic properties, cannot interact with P-gp. Therefore, there is little likelihood of the interaction between P-gp and flavonoids in other tissues. Thanks to numerous studies on cells, it was possible to identify the elements of the flavonoid structure responsible for inhibiting P-gp (Figure 2B). These include hydroxyl groups at the C3 and C5 positions, a double bond between the carbons at C2 and C3, a phenyl substituent at C2, a carbonyl group at C4, and hydrophobic groupings on the A or B ring. Hydrophobicity is essential for P-gp inhibition [69], and the optimal number of hydroxyl groups is three. However, flavonoids with four hydroxyl groups did not show as strong a P-gp inhibitory activity as did the flavonoids with more groups [70].

The curcumin molecule is composed of two parts: a seven-atom linker with a β-diketone in the middle and aromatic rings that are located at the ends of the chain. The same main structures are found among two other P-gp inhibitors: tetrahydrocurcumin and verapamil (Figure 2C). It is suggested that the diketone structure is not necessary to provide multidrug resistance reversal activity in cancer treatment, and in addition, compounds with stronger activity have shorter linkers [71,72].

In the case of alkaloids, in silico studies have shown that piperine can interact with P-gp at both the drug-binding site and NBD, causing its inhibition [73]. Hydrophobic interactions with P-gp, specifically with Leu339, Met69, Met986, Phe72, Phe336, Phe728, Phe983, Tyr953, Val982, and hydrogen bonds with Tyr307 are responsible for its inhibition [73,74]. A structural similarity has been noted between piperine’s 1,3-benzodioxol ring system and 4-chloro-7-nitrobenzofurazan, which is an inhibitor of P-gp that forms bonds with magnesium ions at the ATP-binding site (Figure 2D).

It has been shown that both structures having two electron acceptor groups can interact with divalent cations. The alkenyl side chain of piperine may have some effect on reducing the affinity compared to 4-chloro-7-nitrobenzofurazan, which has additional C=N and NO_2_ groups [75]. Some phytochemicals having a 1,3-benzodioxol ring, including piperine, showed inhibitory effects on both P-gp and CYP3A4 [76].

Studies also have shown a high correlation between the amount of hydrogen bonds formed by the structural elements of berberine and its metabolites (berberrubine, thalifendine, demethyleneberberine, jatrorrhizine, columbamine) with P-gp and the affinity for binding to the extracellular part of P-gp. During the early binding stage, hydrophobic and electrostatic interactions are the main determinant, while during the late release stage, electrostatic interactions are of primary importance. Potent substrates for P-gp that are its competitive inhibitors should have high hydrophobicity to dissolve in the hydrophobic medium of the membrane [77]. The structure of berberine, along with the labeled structures responsible for forming each type of bond, is shown in Figure 2E [78].

## 9. The Study Limitations

Despite the comprehensive analysis provided, this study has several limitations that must be acknowledged. Firstly, the in vitro and in vivo studies conducted on animal models may not fully replicate the complexity of human physiology and cancer biology. While these models provide valuable insights, the extrapolation of results to human subjects should be approached with caution. Another limitation is the variability in the bioavailability and metabolism of dietary compounds. Factors such as individual genetic differences, gut microbiota composition, and overall health status can influence the absorption and efficacy of these compounds. This variability can lead to inconsistent results and may affect the generalizability of the findings. Furthermore, this study primarily focuses on a limited range of dietary compounds and supplements. The vast diversity of phytochemicals and their potential interactions with MDR transporters warrant further investigation. Comprehensive studies encompassing a broader spectrum of dietary components are needed to fully elucidate their roles in modulating drug resistance. Lastly, the potential side effects and toxicity of combining dietary supplements with chemotherapeutic agents were not extensively addressed. While certain supplements may enhance drug efficacy, they may also pose the risks of adverse interactions and toxicity. Future research should aim to establish safe and effective dosages and combinations for clinical use.

In summary, while this study provides significant insights into the influence of diet and dietary supplements on MDR transporters and anticancer therapy, further research is needed to validate these findings in clinical settings and expand the scope of dietary compounds investigated and the wide range of used anticancer drugs.

## 10. Conclusions

**Figure 3:**
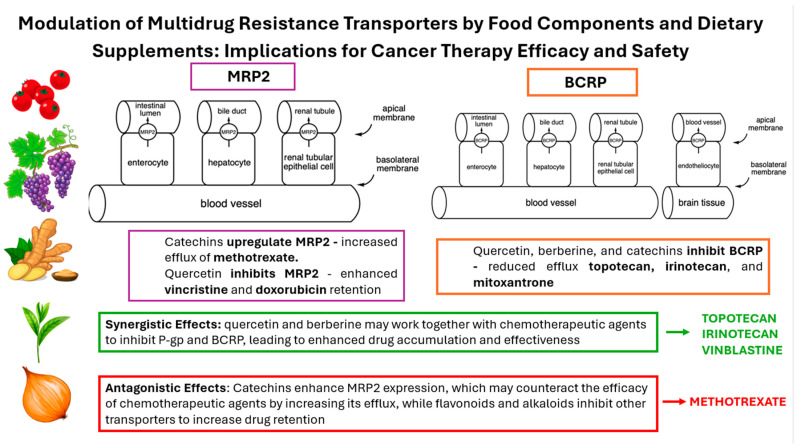
The summary of potential effects of interactions between MDR transporters and selected natural compounds on chemotherapy efficacy.

The findings of this study underscore the critical role of dietary modifications in enhancing the efficacy of chemotherapy (Figure 3). Our results indicate that specific dietary interventions can significantly impact the outcomes of chemotherapy treatments, suggesting that diet should be considered an integral component of cancer care.

Key implications of our research include the following:

- Enhanced efficacy through targeted nutrients: Incorporating a diet rich in specific nutrients, e.g., antioxidants, has been shown to improve specific aspects of chemotherapy efficacy, e.g., tumor response in in vitro or animal studies. However, patients undergoing chemotherapy should be careful in increasing the intake of these nutrients, especially when taken as dietary supplements, and consult healthcare professionals.
- Potential dietary modifications: Based on our findings, we recommend that patients should discuss the possibility of dietary modifications with their oncologists with the help of a clinical pharmacist and clinical dietician. These changes could potentially optimize the effectiveness of chemotherapy and improve overall patient well-being.
- Personalized dietary plans: It is essential for dietary recommendations to be tailored to the individual patient, considering factors such as specific type of cancer, treatment regimen, and personal health conditions. Personalized dietary plans should be developed in collaboration with nutritionists and healthcare providers to ensure the best possible outcomes.

In conclusion, integrating targeted dietary modifications into chemotherapy regimens holds promise for enhancing treatment efficacy and supporting patient health. Future research should continue to explore these dietary interactions to further refine recommendations and improve cancer treatment protocols.

## Data Availability

Data availability on the request.
